# Assessing Motor Variability during Squat: The Reliability of Inertial Devices in Resistance Training

**DOI:** 10.3390/s24061951

**Published:** 2024-03-19

**Authors:** Fernando García-Aguilar, Miguel López-Fernández, David Barbado, Francisco J. Moreno, Rafael Sabido

**Affiliations:** Sport Sciences Department, Miguel Hernandez University, 03202 Elche, Spain; fernando.garciaa@umh.es (F.G.-A.); m.lopezf@umh.es (M.L.-F.); dbarbado@umh.es (D.B.); rsabido@umh.es (R.S.)

**Keywords:** strength training, variability, inertial sensors, non-linear measures, motor control

## Abstract

Movement control can be an indicator of how challenging a task is for the athlete, and can provide useful information to improve training efficiency and prevent injuries. This study was carried out to determine whether inertial measurement units (IMU) can provide reliable information on motion variability during strength exercises, focusing on the squat. Sixty-six healthy, strength-trained young adults completed a two-day protocol, where the variability in the squat movement was analyzed at two different loads (30% and 70% of one repetition maximum) using inertial measurement units and a force platform. The time series from IMUs and force platforms were analyzed using linear (standard deviation) and non-linear (detrended fluctuation analysis, sample entropy and fuzzy entropy) measures. Reliability was analyzed for both IMU and force platform using the intraclass correlation coefficient and the standard error of measurement. Standard deviation, detrended fluctuation analysis, sample entropy, and fuzzy entropy from the IMUs time series showed moderate to good reliability values (ICC: 0.50–0.85) and an acceptable error. The study concludes that IMUs are reliable tools for analyzing movement variability in strength exercises, providing accessible options for performance monitoring and training optimization. These findings have implications for the design of more effective strength training programs, emphasizing the importance of movement control in enhancing athletic performance and reducing injury risks.

## 1. Introduction

Muscular force in the field of physical activity is understood as the capacity of a muscle to produce tension when activated [1]. As adequate force levels present benefits in terms of both athletic performance [2,3] and health [4,5,6], strength exercises are commonly introduced in sport, fitness, and rehabilitation training programs. In order to maximize the benefits and decrease the risks of these strength interventions, most studies have manipulated important parameters related to the exercise load, such as volume, frequency, intensity, etc. [7,8,9]; however, there is a lack of knowledge about how movement control during strength exercises influences the adaptations caused by these programs. 

Movement control, understood as the ability of the nervous and musculoskeletal system to regulate and direct motor actions, exhibits an inherent variability during human movement [10] that seems to reflect how each individual copes with the different task constraints [11]. During strength exercises, movement variations depend on how force is produced and controlled. Force control is the ability to generate accurate and task-relevant force levels and is an important performance factor [12]. During strength exercises, this control is characterized by complex motor fluctuations, which reflect how the different body systems (nervous, musculoskeletal, etc.) adapt motor performance quickly and accurately in response to exercise constraints [13]. These fluctuations are produced at different levels, from the central level, such as recruitment, firing frequency, or coordination of muscle groups, to the effects of peripheral aspects, such as metabolite accumulation [14,15,16,17]. The resultant force output produced during any strength exercise constantly fluctuates, reflecting the continuous interaction between individuals and the task demands [18]. Therefore, movement variability during strength exercise not only depends on each individual’s features (e.g., skill level, current physical condition, experience, etc.), but is also influenced by different constraints such as fatigue [19,20,21] or load [22,23,24], among others.

Non-linear measures have been proposed as relevant tools to analyze movement variability in order to understand how individuals cope with task demands [12]. Specifically, non-linear tools, such as entropy measurements or detrend fluctuation analysis (DFA), have been implemented to describe motor variability during force production tasks [25,26,27]. Entropy parameters and DFA analyze specific aspects of the variability structure, such as signal regularity and fractality, respectively. These tools have been revealed as useful in detecting changes in force control caused by the manipulation of relevant constraints such as fatigue or load magnitude [18,25]. In addition to force control, postural control was another variable where non-linear measures were applied to determine the effect of conditioning factors such as fatigue in lower limb training [28,29]. However, such studies have been conducted on non-functional tasks, which were either single-joint or unrepresentative sporting or everyday actions (e.g., finger press or knee extension). They have also been studied in laboratory settings, which makes it difficult to extrapolate these results to a training context. Studies analyzing common strength exercises such as the squat have focused on kinematic and kinetic variables related to velocity and power [30] through portable biomechanics IMU; however, no work has studied whether the analysis of movement fluctuations during regular exercises such as the squat can be measured reliably to provide useful information for the planning of strength training.

To the best of the authors’ knowledge, no previous research has systematically investigated the reliability of non-linear measures to assess global strength actions using affordable materials. This study aims to fill this gap by examining the reliability of analyzing acceleration signals obtained from accelerometers, specifically in the context of a global movement, involving different joints and the coordination of large muscle groups (e.g., a squat). Our investigation also encompasses assessment of the impact of IMU placement and recording frequency on the reliability of non-linear tools. Furthermore, we explore whether the outcomes of these measurements align with those obtained from a widely used laboratory instrument in sports science, such as a force platform. The significance of this research lies in its potential to establish reliable analysis protocols, which, if validated, can be utilized to investigate the impact of various conditioning factors, including fatigue, loading effects, movement speed, and more. 

## 2. Materials and Methods

### 2.1. Participants

Eighty-eight healthy young people were initially recruited to participate in the study. Seventeen participants did not complete all measures sessions and were therefore eliminated from further analysis. In addition, for five participants, the records were not obtained correctly and, therefore, could not be analyzed. The final sample consisted of 66 participants, and the descriptive data for the participant set are as follows: 34 males (age = 25.7 ± 4.4 years; height = 174.5 ± 7.4 cm; body mass = 71.7 ± 13.6 kg; one repetition maximum (1RM) in the squat = 116.5 ± 23.2 kg; ratio 1RM/body mass = 1.5 ± 0.2) and 32 females (age = 25.1 ± 5.4 years; height = 160.9 ± 5.3 cm; body mass = 60.4 ± 7.6 kg; 1RM in squat = 76.5 ± 17.4 kg; ratio 1RM/body mass = 1.2 ± 0.2). To ensure that all participants had a stable technique, and to avoid affecting reliability results (e.g., through the learning effect), participants were preferred to have at least one year of strength training experience, including the squat exercise in their training programs. To be included in the study, participants completed a health history questionnaire, guaranteeing that they were free from any disease, illness, or injury that may affect the results of the study. Participants were instructed to maintain their normal lifestyle, including nutritional and hydration states. Caffeine intake was not allowed in the 3 h previous to measurements. In addition, strength training sessions were not allowed in the 72 h previous to the experimental sessions. To avoid experimental variability, participants were scheduled at the same time for each session. All participants attended three testing sessions separated by at least 72 h. Prior to participation, each subject provided written informed consent, which was approved by the ethics committee of the University (PID2019-109632RB-100) and which adhered to the Declaration of Helsinki.

### 2.2. Procedures

#### 2.2.1. Day 1—RM Estimation

**Figure 1:**
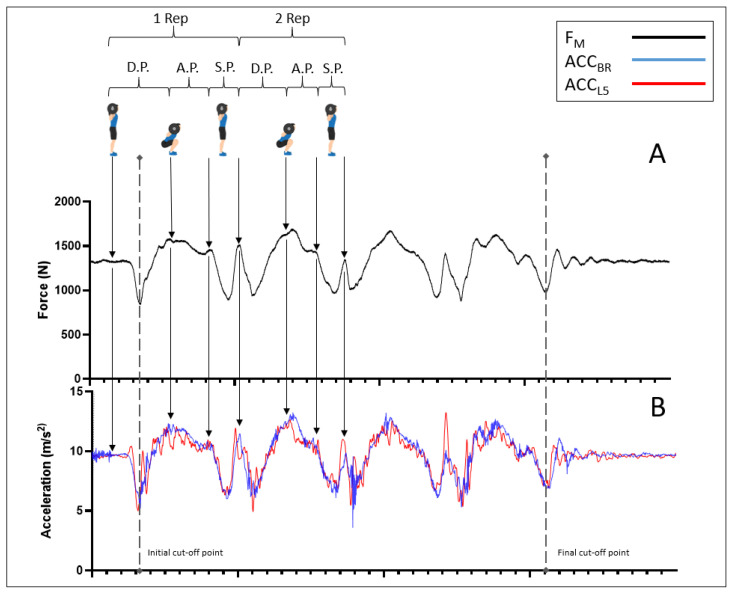
Example of a set of four squats showing the kinetic and kinematic dynamics during the intensity of 70% of the repetition maximum. Illustrated here are the synchronized force and acceleration profiles. The upper graph (**A**) shows the force magnitude (F_M_, in Newton), represented by the solid black line. The lower graph (**B**) displays the acceleration magnitude (m/s^2^), with the blue line representing acceleration at the barbell (ACC_BR_) and the red line indicating acceleration at the lumbar region (ACC_L5_). Dashed vertical lines represent the initial and final cut-off points for data analysis. The phases of the squat movement—ascending (A.P.), descending (D.P.), and stabilization (S.P.)—dotted arrows indicate phase changes approximately. These arrows also relate the graph of F_M_ to that of acceleration in ACC_BR_ and ACC_L5_. Additionally, icons at the top illustrate key positions within the squat cycle: standing and the lowest squat point.

During their first session, participants were familiarized with the warm-up protocol and performed the 1RM squat test. For this 1RM squat test, participants started from a shoulder-width stance apart with the barbell resting on the upper back, approximately at the level of the acromion, with the knees and hips fully extended. Each participant descended until his thighs were parallel to the ground and, subsequently, ascended to the upright position (Figure 1). Participants were encouraged to return to the upright position at maximum speed. The 1RM estimation was automatically calculated by the specialized software of the linear position transducer (T-Force System, V. 3.70, Ergotech, Murcia, Spain). Several studies have supported the use of movement velocity for 1RM estimation [31,32,33]. 

#### 2.2.2. Days 2 and 3—Experimental Procedure

During the second and third sessions, participants performed the experimental protocol, which consisted of a total of two sets of four consecutive repetitions in the squat exercise. Participants performed two sets with 30% and 70% of 1RM, respectively, at a preferred velocity, with a rest period of 4 min between the two situations. All repetitions were completed in all sets.

### 2.3. Data Acquisition

**Figure 2:**
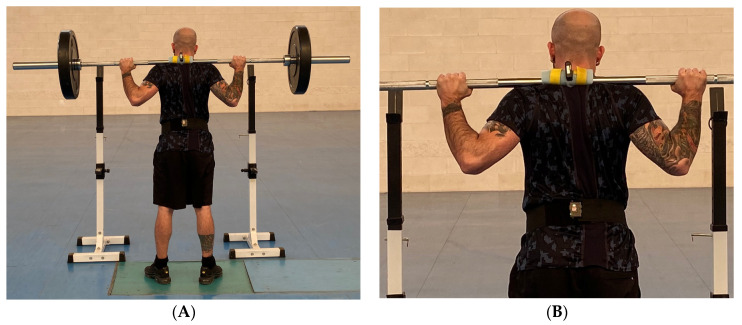
Equipment setup: (**A**) overview; (**B**) placement of the IMUs: one located at the bar and one at L5.

A linear encoder T-ForceTM (T-Force Dynamic Measurement System, Ergotech, Murcia, Spain) was used to calculate RM and monitor mean propulsive velocity during repetitions. During the experimental protocol, the force data were obtained using a Kistler force platform (Winterthur, Switzerland, Mode 9287BA), which was calibrated with InstaCal software V. 7.62 (Measurement Computing Corporation, Norton, MA, USA) before the start of the protocol. The acceleration signal was obtained through two IMUs, which formed part of an inertial motion capture system (iSen, STT Systems Inc., San Sebastián, Spain). One of them was placed in the middle of the barbell with which squats were performed, and the other was placed in the lumbar region at the level of the lumbar vertebra 5 (L5) (Figure 2). The IMUs were synchronized with the iSen software, V. 2023.0. Synchronization between the force platform and the accelerometers was achieved by means of a trigger. The trigger was made by hitting the force platform with an IMU used exclusively for this purpose. Both the IMUs and the force platform recorded at a frequency of 200 Hz. 

### 2.4. Data Analysis

The acceleration magnitude of each of the devices was calculated from the three acceleration axes using the following Equation (1):

(1) $$\sqrt{\left({AP}^{2}+{ML}^{2}{+V}^{2}\right)}$$

where AP is the anterior–posterior axis; ML is the medial–lateral axis; and V is the vertical axis. Thus, the acceleration magnitude of the IMUs located at the barbell (ACC_BR_) and at L5 (ACC_L5_) was obtained. From the force platform, three axes were also obtained: AP, ML, and V. With these axes, F_M_ was calculated using Equation (1). On the other hand, the center of pressure magnitude (COP_M_), which is the resultant of the moments of force in the medial–lateral and anterior–posterior axis, was calculated using the following Equation (2):

(2) $$\sqrt{\left({Ax}^{2}+{Ay}^{2}\right)}$$

where Ax refers to the medial–lateral component of the center of pressures, and Ay to the anterior–posterior component from the force platform.

All of the aforementioned processing steps were performed using an application created ad hoc with LabView 2012 (National Instruments, Austin, TX, USA). In addition, the same application was used to perform the cuts in the time series. The F_M_ module was used to detect the beginning of the first squat and the end of the last squat. The initial cut-off point was the point of lowest force in the first squat, and the final cut-off point was the point of lowest force in the last squat (Figure 1). The original length, i.e., with the recording frequency at 200 Hz, was between 874 and 4703 data. Signals were then sub-sampled at 100 and 50 Hz. Sub-sampling was executed by proportionally adjusting the sampling frequency through the selection of points at regular intervals. To reduce from 200 Hz to 100 Hz, one out of every two points was selected, whereas to decrease to 50 Hz, one out of every four was taken, thus ensuring an adequate temporal representation in the subsampled series. This way, analyses were repeated at three different sampling frequencies (200, 100, and 50 Hz). These frequencies were selected on the basis of practical applicability, as most smartphones integrate accelerometers that work at these frequencies. Thus, the results obtained from this work will be applicable to most devices. The amount of variability was calculated using the standard deviation. The structure of the variability of the time series was analyzed. For this purpose, the time series data were analyzed using fuzzy entropy (FuEn) and sampled entropy (SaEn) to analyze regularity or predictability. These two measures were chosen as they have been shown to be more solid to changes in data length, and FuEn has been shown to be more robust to changes in r and noise [34]. Long-term correlations were also analyzed to provide an indicator of the roughness of the movement, for which DFA was used, as it has been shown to be less affected by the signal’s non-stationarity [35]. FuEn was calculated using a protocol set out by Chen et al. [36], and SaEn was computed based on Yentes et al. [37]. The parameters m = 2, r = 0.2 × SD, and n = 2 [38] were used to calculate FuEn, and m = 2 and r = 0.2 × SD to calculate SaEn [37]. DFA was calculated according to Peng et al. [35] and using windows of one-second duration. For this purpose, the duration of the windows was adjusted according to the sampling frequency. Thus, at 200 Hz, the initial window was 8 data and the final window was 200 data. For 100 Hz, 4 and 100 data were used, respectively, while for 50 Hz, 4 and 50 data were used. The variability analyses performed in this work were carried out using a code that we developed using the Python programming language. 

### 2.5. Statistical Analysis

The obtained data were analyzed using SPSS (V. 25, IBM Statistics, New York, NY, USA). The normality of the data was confirmed using the Kolmogorov–Smirnov test. Relative reliability was assessed through the intraclass correlation coefficient (ICC) [39]. In this analysis, day 2 was compared with day 3 for each of the variables (SD, DFA, FuEn, and SaEn), and for ACC_BR_, ACC_L5_, F_M_ and COP_M_. For the interpretation of these values, we followed Koo and Li [40], who considered an ICC of >0.90 as excellent, 0.75–0.90 as good, 0.50–0.75 as moderate, and <0.50 as poor. Additionally, the absolute reliability was computed for each of the variables in each device using the standard error of measurement (SEM) as the SD of the difference between participants’ trials divided by √2 [41]. This SEM was used to account for the impact of sample heterogeneity and the influence of systematic errors. In this study, the SEM is expressed in absolute values in order to establish what amount of error we can assume in each of the variables. In addition, two complementary analyses were performed. Pearson’s correlation coefficient was calculated to determine the level of correlation between the variables obtained from the force platform and the accelerometers. A two-way ANOVA (intensity and days) was also performed to determine whether the variables studied were sensitive to variations produced by an external factor, in this case, the load. The Bonferroni adjustment was used in the post hoc analysis.

## 3. Results

**Figure 3:**
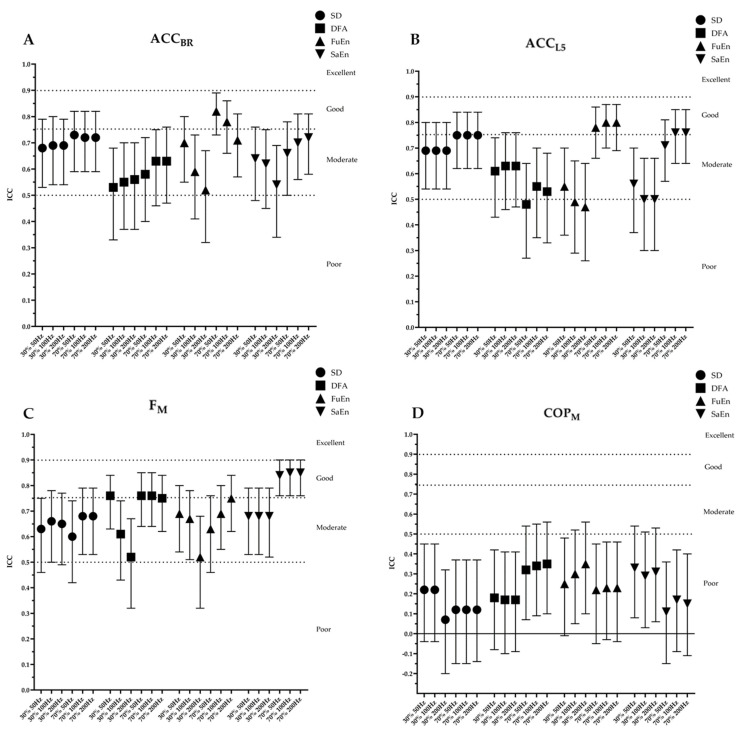
Results of the intraclass correlation coefficient by devices: (**A**) acceleration from the IMU placed on the bar; (**B**) acceleration from the IMU placed on the L5 zone; (**C**) magnitude of the force obtained from the force platform; (**D**) magnitude of the center of pressures obtained from the platform. The graph shows the mean and the upper and lower limits of the 95% confidence interval. The dashed lines indicate the limits for each of the interpretations of the ICC values.

**Figure 4:**
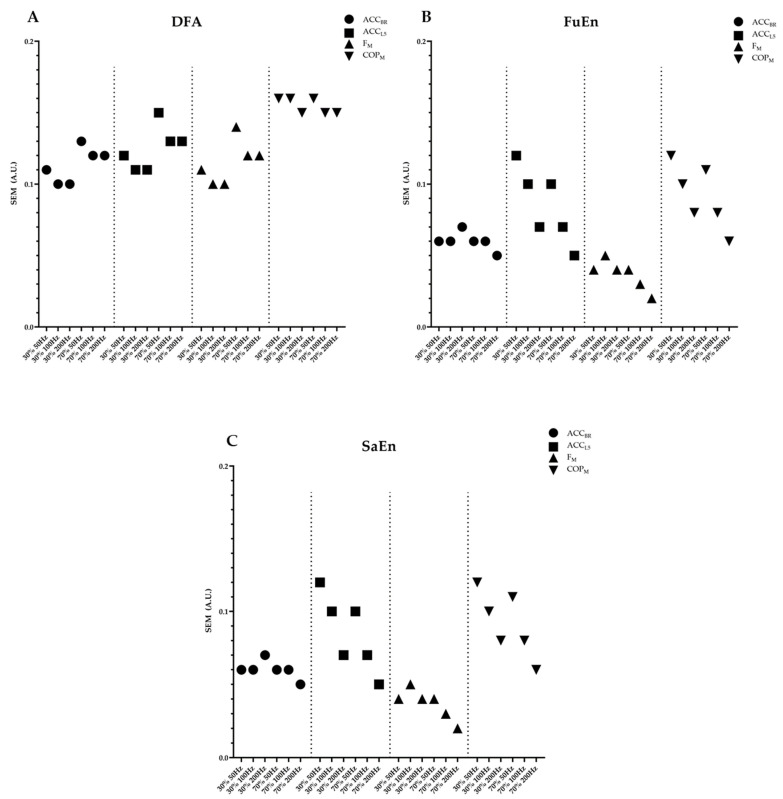
Results of standard errors of measurement by variables: (**A**) detrended fluctuation analysis; (**B**) fuzzy entropy; (**C**) sample entropy. The dashed lines indicate the groupings of the different devices. Note that the SEM standard deviation is not shown in the graphs because given the differences in the measurement magnitudes of each device they are not comparable.

Figure 3 and Figure 4 present a summary of the results obtained in the ICC for each device and the SEM for the non-linear variables, respectively. The SEM values of the SD are measured in different units. In summary, in terms of ICC, most of the outcomes calculated from IMUs and F_M_ showed moderate to good ICC values, but COP_M_ did not show acceptable ICC values for any variable. The impact of frequency on ICC was minimal, as the differences were generally less than 0.1 for most measures and variables. SEM is shown with absolute values, in m/s^2^ for SD and unitless for non-linear variables. A more detailed description of the results shown in each table follows. 

Table 1 and Table 2 present the mean values and standard deviation together with the reliability results of the time series obtained from the IMUs placed at the barbell and at L5 area, respectively. The ACC_BR_ showed a moderate to good ICC, with values ranging from 0.52 to 0.82. The ICC values at 70% load were slightly higher than at 30% load. Regarding SEM, the following values were observed for each variable: SD ranged from 0.33 to 0.49, DFA ranged from 0.10 to 0.13, FuEn ranged from 0.05 to 0.07, and SaEn ranged from 0.05 to 0.10. The results are similar to those obtained for ACC_L5_. For the ICC value, the range for ACC_L5_ was from 0.47 to 0.80. It appears that the trend towards higher ICC values in the 70% load compared to the 30% load was maintained in ACC_L5_ for all variables except DFA. Regarding SEM, ACC_L5_ reported the following values: SD from 0.30 to 0.44, DFA from 0.11 to 0.15, FuEn from 0.07 to 0.12, and SaEn from 0.05 to 0.14.

Table 3 and Table 4 show the mean values and standard deviations together with the reliability results of the time series F_M_ and COP_M_, respectively. The two types of time series were obtained from the force platform. In the same way as IMUs, the F_M_ ICC values were also moderate to good (from 0.52 to 0.85). And the SEM values had SDs between 37.22 and 47.16, DFA between 0.10 and 0.14, FuEn between 0.02 and 0.05, and SaEn between 0.03 and 0.07. Contrary to the other measures, the ICC scores for COPM outcomes were low for all variables (ICC < 0.35). With respect to SEM, the values reported for COP_M_ were 5.48–9.67 for SD, 0.15–0.16 for DFA, 0.06–0.12 for FuEn, and 0.07–0.12 for SaEn.

The results of Pearson correlation are shown in Table 5. The acceleration values from the IMUs showed strong correlations between SD (0.94 < r < 0.98) and DFA (0.74 < r < 0.83) in both load intensity conditions. Entropy measures exhibited correlations ranging from weak (0.23 < r < 0.39) for 30% RM to strong (0.63 < r < 0.79) for 70% RM. The correlation values between F_M_ and the IMUs ranged from −0.17 to 0.96 (F_M_-ACC_BR_: −0.17–0.096; F_M_-ACC_L5_ sacrum: 0.18–0.82). In F_M_, a strong correlation was reported with both ACC_BR_ and ACC_L5_ for SD and DFA (0.71 < r < 0.96) for both intensities. In the entropy measurements, the correlation was weak to moderate (−0.17 < r < 0.48) for both ACC_BR_ and ACC_L5_ with F_M_. Regarding COP_M_ correlations, no significant results were obtained in the analysis of the remaining variables.

The ANOVA revealed significant differences (*p* < 0.05) between the 30% and 70% RM conditions for different devices and variables. Notably, there were no significant differences between days, except for the FuEn of ACC_L5_ and SD in COP_M_, although the trends in both measures were similar. It is worth noting that the trends resulting from increasing intensity only aligned with DFA, where an escalation in intensity corresponded to a decrease in DFA values. In the remaining variables, trends diverged between measurements obtained from the F_M_ and COP_M_ compared to those from the IMUs, encompassing both ACC_BR_ and ACC_L5_. For example, while SD increased with a higher load in the F_M_ and COP_M_ measurements, it decreased in the IMUs. Conversely, in the 70% RM condition, FuEn and SaEn values decreased in the F_M_ and COP_M_ but increased in the IMU, compared to the 30% RM condition.

## 4. Discussion

This study was conducted to investigate whether movement variability measured through inertial sensors can provide reliable information on movement control during a strength exercise such as the squat. Specifically, on the one hand, we assessed relative reliability through the ICC to determine whether participants could be classified properly according to their movement variability. On the other hand, we analyzed absolute reliability to define the range threshold that can help to determine whether changes in movement variability during the squat are caused by individuals´ inherent variability or an external factor (i.e., learning, adaptation, fatigue, etc.).

Firstly, the ICC was used to determine the relative reliability and consistency of the measurements. A high ICC suggests consistency and agreement between different measurements, indicating that it allows for ranking [41]. While the ACC_BR_ and ACC_L5_, together with the F_M_, showed acceptable to good values for practically all variables, the COP_M_ showed the lowest reliability values, as the ICC did not reach an acceptable threshold in any case. We have not found studies that have analyzed the relative reliability of movement control variability in strength tasks, whether with linear or non-linear measurements. To provide some references, we can examine related works. When assessing relative reliability using the ICC in strength tasks with accelerometers measuring variables such as velocity, power, or force, reported results have ranged from good to excellent in most of the studies, and only one of the studies reviewed showed poor ICC values in velocity variables [30]. It is noteworthy that in ACC_BR_, ACC_L5_, and F_M_, ICC values are slightly higher at higher loads for most variables. The only exception to this trend is observed in DFA of ACC_L5_. This might suggest that higher loads pose a greater challenge, consequently allowing for better classification. Regarding the COP_M_, although some studies have shown the reliability of non-linear tools in balance tasks, with ICC values between acceptable and good [42,43,44,45,46], our results suggest that, in strength tasks, they are not reliable (ICC < 0.50). We propose several explanations. The first is the difference in postural adjustments that occur when performing a dynamic task with weight, such as a squat, and those that occur when performing a static balance task with body weight only. Another reason for this may be the greater non-stationarity of the squat signal compared to the balance signals. Finally, in terms of frequency, the differences are minimal, typically less than 0.1 units, indicating that recording between frequencies of 50 and 200 Hz does not appear to affect relative reliability. With this in mind, it can be suggested that ACC_BR_, ACC_L5_, and F_M_ show acceptable relative reliability, indicating that motion analysis involving linear and non-linear variables can be measured consistently, allowing for the classification of subjects.

Secondly, to determine the precision of the measurement, the SEM was used to quantify the absolute reliability. The SEM makes it possible to define the range within which the true value of the measurement should lie [47], and, thus, to determine whether the changes (or lack thereof) are an effect of the intervention or caused by random errors in measurement. It is important to note that the SEM depends on the magnitude of the measurement. In other words, if a measurement yields large values, a larger SEM can be accepted, and vice versa. For instance, the acceptability of an SEM for DFA is different from that for SD when the measurement values differ significantly. The ranges of SEM are different between variables in SD; in IMUs, the SEM is between 0.30 and 0.49; in F_M_, the range is between 37.22 and 47.16; and in COP_M_, it ranges between 5.48 and 9.67. In non-linear measurements, the values are more similar between ranges of 0.30 and 0.49. Thus, the SEM complements the reliability information provided by the ICC, offering an index of the variations required in measurements to determine whether a change resulting from an intervention is genuinely due to that intervention and not merely a random error. Considering that the SEM is contingent on the type of measurement, and given the limited literature on variability in acceleration signals during strength actions, we are unable to directly compare the precision of these measures. However, based on the descriptive data presented in Table 1, Table 2, Table 3 and Table 4, it can be suggested that these measures are capable of detecting changes. However, when comparing SEM values in FuEn and SaEn, given their similar nature, minimal differences are observed (never exceeding 0.05), suggesting a comparable margin of error in both measurements. On the other hand, DFA also exhibits SEM values relatively close to those of entropy measures. Considering that DFA values tend to be higher, DFA is likely a more robust measure against measurement error. Across all variables, the SEM is consistently smaller than the between-subject standard deviation, implying that the measurement is responsive to changes. Previous studies have reported variations in SEM values for non-linear variables. Lin et al. [44] reported lower SEM values (0.04 < SEM < 0.06) in DFA during balancing tasks, while Mazaheri et al. [48] observed greater SEM ranges (0.20 < SEM < 0.37) in entropy measurements, also in the context of balancing tasks. This divergence in SEM values across studies employing similar tasks could be attributed to differences in the measurements (DFA versus entropy). When we compare these findings with our research, it appears that SEM values may fluctuate based on the nature of the task, even when the analysis is performed through analogous variables. These discrepancies could be due to factors similar to those mentioned above for the ICC, including task-specific adjustments, non-stationarity, and variances in the variable’s value range. Importantly, the SEM provides critical insight into the expected measurement error, offering valuable information for interpreting the reliability and precision of the data.

Additionally, frequency is an important factor that can modify entropy and DFA values [49,50]. For this reason, we compared the results of the non-linear measures at different frequencies. The reported findings indicate that, while there are variations in the absolute values when changing frequencies, the same trends persist. Similar results are presented in the study by Caballero et al. [51], whereas in our study, an increase in SaEn values and a decrease in DFA values were reported with increasing frequency, but without significantly influencing the results. Furthermore, the reliability values (ICC, SEM) remained consistent. This suggests that different frequencies can be used interchangeably. For the analyses to be valid, the frequency must be adjusted to the movement and/or process to be analyzed [50,51]. However, it should be noted that if we wish to compare absolute values, it is essential to compare values within the same frequency.

The correlation analysis revealed strong correlations between the two IMUs and between the F_M_ and each of the two IMUs in the variables of SD and DFA. By contrast, for the FuEn and SaEn variables, a strong correlation (r > 0.50) was reported between the two IMUs at the 70% RM load condition, while a weak correlation was observed at the 30% RM load condition. Meanwhile, correlations between the IMUs and the F_M_ varied from moderate to non-existent. The COP_M_ showed weak or non-existent correlations with all measures. Other studies have analyzed the validity of accelerometers by comparing them with gold standards such as the center of pressure (COP) [52,53] motion capture systems [54] or gait analysis systems [55], reporting moderate to high correlation values. Our results differ in terms of COP, as in no case do the correlations reach moderate. It is possible that this is due to differences in the task (squat vs. balance) mentioned above. Nevertheless, we observed moderate to high correlations between force modulus and IMUs, particularly in dynamic actions with substantial force requirements. This suggests that both F_M_ and IMUs can effectively capture the variability structure in tasks such as the squat. Furthermore, it relates body oscillations, reflected in acceleration, to fluctuations in force production, reflected in F_M_.

Finally, the conducted ANOVA demonstrated differences between load conditions in all variables and measures. This suggests that these protocols are sensitive to changes in load. Additionally, the absence of differences between days in the majority of variables, and when differences exist, observing consistent trends, further reflects the day-to-day validity of these measures.

## 5. Conclusions

The main objective of this study was to assess the reliability of different measures of variability in a strength movement such as the squat. While we successfully achieved this goal, contributing valuable insights, it is important to acknowledge certain limitations. Firstly, given that the study focused on a single task (squat) and modified a single factor (load), the results should be approached with caution when extrapolating them to other tasks or conditions. Therefore, further research is needed to investigate these measures in tasks with additional independent variables such as a broader range of loads, fatigue, level of expertise, etc. Additionally, consideration should be given to the characteristics of the time series, which are of variable length and non-stationary. Lastly, when interpreting results and drawing conclusions, the lack of literature addressing similar tasks poses a challenge. Therefore, continued research is essential to establish a robust knowledge base in this field.

Both force and acceleration magnitude, whether measured on the barbell or close to the L5 area, are reliable variables for assessing variability in tasks involving substantial force, such as squats. However, the use of the COP_M_ is not recommended for this purpose. The most robust measure across all three devices is DFA, as it consistently yields results ranging from acceptable to good across the two reliability metrics employed in this study (ICC and SEM). The choice of a sampling frequency between 50 and 200 Hz seems to have had no significant impact on the relative results, although it did affect absolute values. Furthermore, these measures can be used interchangeably with both the magnitude force and IMUs, making them accessible to a wider range of users.

## Figures and Tables

**Table 1:** Descriptive and reliability results of linear and non-linear parameters obtained from the acceleration magnitude time series recorded from IMU placed at the barbell.

Variable	%RM	Frequency	Day 2	Day 3	Reliability Measures	
			M ± SD	M ± SD	ICC (LCL-UCL)	SEM
SD	30	50	2.60 ± 0.90	2.72 ± 0.84	0.68 (0.53, 0.79)	0.49
		100	2.60 ± 0.89	2.72 ± 0.83	0.69 (0.54, 0.8)	0.48
		200	2.60 ± 0.89	2.72 ± 0.83	0.69 (0.54, 0.79)	0.48
	70	50	2.11 ± 0.62	2.15 ± 0.61	0.73 (0.59, 0.82)	0.33
		100	2.13 ± 0.63	2.15 ± 0.61	0.72 (0.59, 0.82)	0.33
		200	2.14 ± 0.63	2.16 ± 0.61	0.72 (0.59, 0.82)	0.33
DFA	30	50	1.29 ± 0.18	1.32 ± 0.15	0.53 (0.33, 0.68)	0.11
		100	1.33 ± 0.17	1.34 ± 0.14	0.55 (0.37, 0.70)	0.10
		200	1.32 ± 0.17	1.34 ± 0.14	0.56 (0.37, 0.70)	0.10
	70	50	1.14 ± 0.22	1.18 ± 0.2	0.58 (0.4, 0.72)	0.13
		100	1.19 ± 0.20	1.21 ± 0.18	0.63 (0.46, 0.75)	0.12
		200	1.18 ± 0.20	1.21 ± 0.18	0.63 (0.47, 0.76)	0.12
FuEn	30	50	0.54 ± 0.12	0.54 ± 0.10	0.70 (0.55, 0.80)	0.06
		100	0.32 ± 0.11	0.32 ± 0.09	0.59 (0.41, 0.73)	0.06
		200	0.19 ± 0.10	0.19 ± 0.08	0.52 (0.32, 0.67)	0.07
	70	50	0.68 ± 0.15	0.67 ± 0.15	0.82 (0.73, 0.89)	0.06
		100	0.44 ± 0.12	0.43 ± 0.12	0.78 (0.66, 0.86)	0.06
		200	0.26 ± 0.11	0.26 ± 0.10	0.71 (0.57, 0.81)	0.05
SaEn	30	50	0.52 ± 0.14	0.52 ± 0.11	0.64 (0.48, 0.76)	0.08
		100	0.34 ± 0.11	0.34 ± 0.09	0.62 (0.45, 0.75)	0.06
		200	0.22 ± 0.10	0.22 ± 0.08	0.54 (0.34, 0.69)	0.06
	70	50	0.59 ± 0.15	0.57 ± 0.19	0.66 (0.5, 0.78)	0.10
		100	0.39 ± 0.12	0.39 ± 0.14	0.70 (0.56, 0.81)	0.07
		200	0.26 ± 0.09	0.25 ± 0.10	0.72 (0.58, 0.81)	0.05

Note. %RM: RM percentage; M: mean; SD: standard deviation; ICC: intraclass correlation coefficient; LCL: lower confidence interval; UCL: upper confidence interval; SEM: standard error of measurement; DFA: detrended analysis fluctuation; FuEn: fuzzy entropy; SaEn: sample entropy.

**Table 2:** Descriptive and reliability results of linear and non-linear parameters obtained from the acceleration magnitude time series recorded from IMU placed at the L5.

Variable	%RM	Frequency	Day 2	Day 3	Reliability Measures	
			M ± SD	M ± SD	ICC (LCL-UCL)	SEM
SD	30	50	2.31 ± 0.84	2.42 ± 0.77	0.69 (0.54, 0.8)	0.44
		100	2.32 ± 0.85	2.42 ± 0.77	0.69 (0.54, 0.8)	0.44
		200	2.32 ± 0.85	2.42 ± 0.77	0.69 (0.54, 0.8)	0.44
	70	50	1.95 ± 0.6	2 ± 0.54	0.75 (0.62, 0.84)	0.30
		100	1.96 ± 0.6	2 ± 0.54	0.75 (0.62, 0.84)	0.30
		200	1.96 ± 0.6	2 ± 0.54	0.75 (0.62, 0.84)	0.30
DFA	30	50	1.19 ± 0.21	1.18 ± 0.18	0.61 (0.43, 0.74)	0.12
		100	1.22 ± 0.2	1.21 ± 0.17	0.63 (0.46, 0.76)	0.11
		200	1.22 ± 0.2	1.2 ± 0.17	0.63 (0.47, 0.76)	0.11
	70	50	0.96 ± 0.2	0.99 ± 0.21	0.48 (0.27, 0.64)	0.15
		100	1 ± 0.19	1.01 ± 0.19	0.55 (0.35, 0.7)	0.13
		200	1 ± 0.19	1.01 ± 0.19	0.53 (0.33, 0.68)	0.13
FuEn	30	50	0.87 ± 0.22	0.84 ± 0.15	0.55 (0.36, 0.7)	0.12
		100	0.55 ± 0.17	0.52 ± 0.12	0.49 (0.29, 0.65)	0.10
		200	0.29 ± 0.11	0.27 ± 0.08	0.47 (0.26, 0.64)	0.07
	70	50	1.09 ± 0.21	1.05 ± 0.21	0.78 (0.66, 0.86)	0.10
		100	0.72 ± 0.16	0.69 ± 0.15	0.80 (0.7, 0.87)	0.07
		200	0.4 ± 0.11	0.38 ± 0.1	0.80 (0.69, 0.87)	0.05
SaEn	30	50	0.89 ± 0.23	0.83 ± 0.17	0.56 (0.37, 0.7)	0.14
		100	0.58 ± 0.17	0.55 ± 0.13	0.50 (0.3, 0.66)	0.11
		200	0.35 ± 0.11	0.33 ± 0.08	0.50 (0.3, 0.66)	0.07
	70	50	1.05 ± 0.24	1.01 ± 0.24	0.71 (0.57, 0.81)	0.13
		100	0.72 ± 0.19	0.69 ± 0.19	0.76 (0.64, 0.85)	0.09
		200	0.43 ± 0.11	0.42 ± 0.11	0.76 (0.64, 0.85)	0.05

Note. %RM: RM percentage; M: mean; SD: standard deviation; ICC: intraclass correlation coefficient; LCL: lower confidence interval; UCL: upper confidence interval; SEM: standard error of measurement; DFA: detrended analysis fluctuation; FuEn: fuzzy entropy; SaEn: sample entropy.

**Table 3:** Descriptive and reliability results of linear and non-linear parameters obtained from the force magnitude time series.

Variable	%RM	Frequency	Day 2	Day 3	Reliability Measures	
			M ± SD	M ± SD	ICC (LCL-UCL)	SEM
SD	30	50	197.89 ± 85.64	207.28 ± 73.48	0.68 (0.53, 0.79)	46.55
		100	198.3 ± 85.9	208.09 ± 73.69	0.68 (0.53, 0.79)	46.86
		200	199.35 ± 85.68	207.87 ± 73.76	0.68 (0.52, 0.79)	47.16
	70	50	240 ± 88	242.29 ± 84.19	0.84 (0.76, 0.9)	37.23
		100	240.75 ± 88.31	242.92 ± 84.44	0.85 (0.76, 0.9)	37.24
		200	241.08 ± 88.47	243.01 ± 84.53	0.85 (0.76, 0.9)	37.22
DFA	30	50	1.27 ± 0.18	1.3 ± 0.17	0.63 (0.46, 0.75)	0.11
		100	1.31 ± 0.18	1.32 ± 0.16	0.66 (0.5, 0.78)	0.10
		200	1.31 ± 0.18	1.32 ± 0.16	0.65 (0.49, 0.77)	0.10
	70	50	1.1 ± 0.23	1.13 ± 0.24	0.60 (0.42, 0.74)	0.14
		100	1.14 ± 0.22	1.17 ± 0.22	0.68 (0.53, 0.79)	0.12
		200	1.14 ± 0.22	1.16 ± 0.22	0.68 (0.53, 0.79)	0.12
FuEn	30	50	0.49 ± 0.09	0.49 ± 0.09	0.76 (0.63, 0.84)	0.04
		100	0.29 ± 0.07	0.28 ± 0.07	0.61 (0.43, 0.74)	0.05
		200	0.15 ± 0.06	0.15 ± 0.06	0.52 (0.32, 0.67)	0.04
	70	50	0.44 ± 0.08	0.43 ± 0.08	0.76 (0.64, 0.85)	0.04
		100	0.27 ± 0.06	0.27 ± 0.06	0.76 (0.64, 0.85)	0.03
		200	0.14 ± 0.05	0.14 ± 0.04	0.75 (0.62, 0.84)	0.02
SaEn	30	50	0.47 ± 0.12	0.47 ± 0.12	0.69 (0.54, 0.8)	0.07
		100	0.31 ± 0.1	0.31 ± 0.1	0.67 (0.51, 0.78)	0.06
		200	0.2 ± 0.08	0.2 ± 0.08	0.52 (0.32, 0.68)	0.06
	70	50	0.35 ± 0.1	0.34 ± 0.1	0.63 (0.46, 0.76)	0.06
		100	0.24 ± 0.08	0.23 ± 0.07	0.69 (0.55, 0.8)	0.04
		200	0.16 ± 0.06	0.15 ± 0.06	0.75 (0.62, 0.84)	0.03

Note. %RM: RM percentage; M: mean; SD: standard deviation; ICC: intraclass correlation coefficient; LCL: lower confidence interval; UCL: upper confidence interval; SEM: standard error of measurement; DFA: detrended analysis fluctuation; FuEn: fuzzy entropy; SaEn: sample entropy.

**Table 4:** Descriptive and reliability results of linear and non-linear parameters obtained from the center of pressure magnitude time series.

Variable	%RM	Frequency	Day 2	Day 3	Reliability Measures	
			M ± SD	M ± SD	ICC (LCL-UCL)	SEM
SD	30	50	18.71 ± 6	16.72 ± 5.42	0.22 (−0.04, 0.45)	5.49
		100	18.73 ± 5.99	16.72 ± 5.42	0.22 (−0.04, 0.45)	5.48
		200	20.57 ± 11.07	16.73 ± 5.43	0.07 (−0.2, 0.32)	9.67
	70	50	22.64 ± 9.89	19.9 ± 6.79	0.12 (−0.15, 0.37)	7.74
		100	22.66 ± 9.89	19.93 ± 6.8	0.12 (−0.15, 0.37)	7.72
		200	22.67 ± 9.9	19.99 ± 6.83	0.12 (−0.14, 0.37)	7.74
DFA	30	50	1.35 ± 0.18	1.38 ± 0.19	0.18 (−0.08, 0.42)	0.16
		100	1.37 ± 0.17	1.4 ± 0.17	0.17 (−0.1, 0.41)	0.16
		200	1.37 ± 0.17	1.4 ± 0.17	0.17 (−0.09, 0.41)	0.15
	70	50	1.28 ± 0.2	1.32 ± 0.17	0.32 (0.07, 0.54)	0.16
		100	1.31 ± 0.19	1.34 ± 0.16	0.34 (0.09, 0.55)	0.15
		200	1.31 ± 0.19	1.34 ± 0.16	0.35 (0.1, 0.56)	0.15
FuEn	30	50	0.48 ± 0.14	0.5 ± 0.14	0.25 (−0.01, 0.48)	0.12
		100	0.28 ± 0.13	0.3 ± 0.12	0.3 (0.05, 0.52)	0.10
		200	0.16 ± 0.12	0.18 ± 0.09	0.35 (0.1, 0.56)	0.08
	70	50	0.45 ± 0.13	0.45 ± 0.13	0.22 (−0.05, 0.45)	0.11
		100	0.24 ± 0.1	0.25 ± 0.09	0.23 (−0.03, 0.46)	0.08
		200	0.12 ± 0.08	0.13 ± 0.06	0.23 (−0.04, 0.46)	0.06
SaEn	30	50	0.52 ± 0.15	0.52 ± 0.15	0.33 (0.08, 0.54)	0.12
		100	0.41 ± 0.14	0.43 ± 0.13	0.29 (0.03, 0.51)	0.12
		200	0.21 ± 0.13	0.22 ± 0.1	0.31 (0.06, 0.53)	0.09
	70	50	0.46 ± 0.12	0.47 ± 0.12	0.11 (−0.15, 0.36)	0.11
		100	0.37 ± 0.12	0.37 ± 0.11	0.17 (−0.09, 0.42)	0.10
		200	0.16 ± 0.08	0.17 ± 0.06	0.15 (−0.11, 0.4)	0.07

Note. %RM: RM percentage; M: mean; SD: standard deviation; ICC: intraclass correlation coefficient; LCL: lower confidence interval; UCL: upper confidence interval; SEM: standard error of measurement; DFA: detrended analysis fluctuation; FuEn: fuzzy entropy; SaEn: sample entropy.

**Table 5:** Correlations between devices.

		ACC_BR_-ACC_L5_	F_M_-ACC_BR_	F_M_-ACC_L5_	COP_M_-ACC_BR_	COP_M_-ACC_L5_	COP_M_-F_M_
%RM	Frequency	Variable					
SD							
30	50 Hz	0.98 **	0.78 **	0.74 **	0.29 *	0.27 *	0.32 *
	100 Hz	0.97 **	0.78 **	0.73 **	0.28 *	0.27 *	0.32 *
	200 Hz	0.97 **	0.78 **	0.73 **	0.28 *	0.27 *	0.32 *
70	50 Hz	0.94 **	0.73 **	0.77 **	0.2	0.19	0.07
	100 Hz	0.94 **	0.73 **	0.77 **	0.18	0.15	0.04
	200 Hz	0.94 **	0.73 **	0.77 **	0.21	0.18	0.07
DFA							
30	50 Hz	0.83 **	0.96 **	0.82 **	0.09	0.03	0.14
	100 Hz	0.82 **	0.95 **	0.82 **	0.13	0.08	0.2
	200 Hz	0.83 **	0.95 **	0.82 **	0.13	0.08	0.2
70	50 Hz	0.78 **	0.94 **	0.75 **	0.28 *	0.23	0.22
	100 Hz	0.75 **	0.96 **	0.71 **	0.32 *	0.29 *	0.26 *
	200 Hz	0.74 **	0.96 **	0.72 **	0.29 *	0.24	0.25
FuEn							
30	50 Hz	0.39 **	0.45 **	0.45 **	0.03	0.07	0.22
	100 Hz	0.28 *	0.06	0.34 **	0.04	0.11	0.22
	200 Hz	0.23	−0.17	0.18	0.03	0.14	0.18
70	50 Hz	0.79 **	0.43 **	0.35 **	0.20	0.23	0.15
	100 Hz	0.65 **	0.31 **	0.29 *	0.19	0.14	0.16
	200 Hz	0.55 **	0.12	0.24 *	0.14	0.14	0.08
SaEn							
30	50 Hz	0.39 **	0.43 **	0.42 **	−0.02	0.14	0.28 *
	100 Hz	0.34 **	0.2	0.36 **	0.02	0.11	0.16
	200 Hz	0.32 **	−0.01	0.25 *	0.07	0.25	0.28 *
70	50 Hz	0.74 **	0.48 **	0.46 **	0.11	0.12	0.11
	100 Hz	0.63 **	0.36 **	0.36 **	0.13	0.09	−0.07
	200 Hz	0.66 **	0.26 *	0.35 **	0.13	0.13	0.1

Note. %RM: RM percentage; SD: standard deviation; DFA: detrended analysis fluctuation; FuEn: fuzzy entropy; SaEn: sample entropy. COP_M_: center of pressure magnitude; F_M_: force magnitude; ACC_BR_: acceleration magnitude from IMU barbell; ACC_L5_: acceleration magnitude from IMU L5; *: *p* < 0.05; **: *p* < 0.01.

## Data Availability

The data that support the findings of this study are available from the corresponding author upon reasonable request. Similarly, the codes used to perform the variability analyses are available upon request to the corresponding author.
